# Three Presentations of Takayasu's Arteritis in Hispanic Patients

**DOI:** 10.1155/2012/839795

**Published:** 2012-11-28

**Authors:** Ramy Magdy Hanna, Wan-Ting Yang, Susan Jene Kim, Eduardo A. Lopez, Joseph Nabil Riad, James Wilson

**Affiliations:** ^1^Department of Medicine, Olive-View UCLA Medical Center, Los Angeles, CA 91342, USA; ^2^The David Geffen School of Medicine at UCLA, Los Angeles, CA 90095, USA; ^3^Division of Nephrology, Olive-View UCLA Medical Center, Los Angeles, CA 91342, USA

## Abstract

Takayasu's arteritis (TA) is a medium and large vessel vasculitis, defined as a nonspecific aortitis that usually involves the aorta and its branches Kobayashi and Numano (2002). Its etiology remains unclear, and its complications are diverse and severe, including stenosis of the thoracic and abdominal aorta, aortic valve damage and regurgitation, and stenosis of the branches of the aorta. Carotid stenosis, coronary artery aneurysms, and renal artery stenosis resulting in renovascular hypertension are also reported sequellae of TA Kobayashi and Numano (2002). The disease was first described in Japan, but has also been diagnosed in India and Mexico Johnston (2002). Its incidence in the United States has been quoted as 2.6 patients per 1,000,000 people/year Johnston (2002). In Japan, its incidence is 3.6 patients per 1,000,000 patients/year and prevalence is 7.85 patients per 100,000 per year Morita et al. (1996). The natural history of this disease, which is commonly present in Asian populations, has only recently been studied in Hispanic patients despite the notable incidence and prevalence of TA in Mexican, South American, and Indian populations (Johnston 2002, Gamarra et al. 2010 ). We present three cases of Hispanic patients who presented with TA at Olive-View-UCLA Medical Center (OVMC). We review their clinical and radiographic presentations. Finally, we review the literature to compare the clinical features of our three patients with data regarding the presentation of TA in more traditional Asian populations.

## 1. Introduction

Takayasu's arteritis (TA) is a rare condition characterized by idiopathic chronic inflammation of the medium and large vessels. It often affects the aorta and its major branches. The disease is characterized by granulomatous inflammation of the aortic branches, leading to stenosis, thrombosis, and aneurysm formation. While the disease more commonly affects females in their second and third decades of life, patients as young as six months of age have been described in the literature. 

The criteria proposed for the clinical diagnosis of Takayasu's arteriopathy were based on clinical and angiographic data from 108 Japanese patients (96 of whom had TA and 12 with other aortic diseases). The criteria consist of one obligatory criterion (age less than or equal to forty years), two major criteria (left and right mid-subclavian artery lesions), and nine minor criteria (high erythrocyte sedimentation rate, common carotid artery tenderness, hypertension, aortic regurgitation or annuloaortic ectasia and lesions of the pulmonary artery, left mid common carotid artery, distal brachiocephalic trunk, thoracic aorta, and abdominal aorta). The presence of 2 major criteria, 1 major plus 2 or more minor criteria, or 4 or more minor criteria suggest a high probability of Takayasu disease (84% sensitivity) [1]. Hypertension due to renovascular compromise is unusual as the only presenting feature of TA [1]. 

 As previously noted, TA is a rare disease in the United States with a cited incidence of 2.6 cases/million patients [2]. The incidence in Japan is 3.6 cases/million per year, and the prevalence of TA in Japan is 7.85 cases/100,000 patients [3]. Its incidence worldwide is pretty uniform at approximately 1 to 2 per million per year [4]. TA typically affects young females of “Mongoloid ancestry” per Hernández Pando et al. [5]. As expected, the prevalence of the disease is higher in Latin Americans as well as populations with Asian ancestry. There is one new case series that examines the prevalence and the clinical characteristics of antineutrophil cytoplasmic antibody (ANCA positive) vasculitides and TA in Central and South American populations (Brazil, Chile, Mexico, Columbia, and Peru) [6]. Until now, there has not been an effort to look at the epidemiology or the typical presentation of TA in Hispanic patients in the United States. A study of patients in Mexico found no significant difference in the clinical presentations between TA patients there and in Japan [7]. Extensive research in Central America did bring to light a causative association not common in Japanese populations of TA patients. This is the newly documented link between *Mycobacterium Tuberculosis* infection and TA [5, 8]. It is well known that TB is very prevalent in South and Central America and this could explain some of the increased prevalence of TA there. New links between ulcerative colitis (UC) and TA have also come to light in case reports of Turkish and Japanese patients [9–11]. It remains to be seen whether these associations will also be found in the Hispanic-American population. 

This case-series examines three patients with TA in the Hispanic population. These patients presented to Olive View-UCLA Medical center (OVMC), a tertiary care UCLA run academic hospital in Los Angeles, California with a predominantly Hispanic patient population. About 50% of TA patients have hypertension [1], and our three cases are no exception. The three cases are described in detail below along with pertinent clinical and radiographical findings.

## 2. Case Presentations

### 2.1. Case Number 1: Patient J.S

Patient J.S. is a 40-year-old woman who was referred to nephrology for treatment of refractory hypertension by her endocrinologist, who was managing her acquired hypothyroidism. The patient initially presented to her primary doctor at age 34 with a right-sided neck mass and workup of that mass revealed a right Hurtle cell adenoma. She was then referred to nephrology for severe hypertension, refractory to years of multiple combination-therapy regimens. 

**Figure 1:**
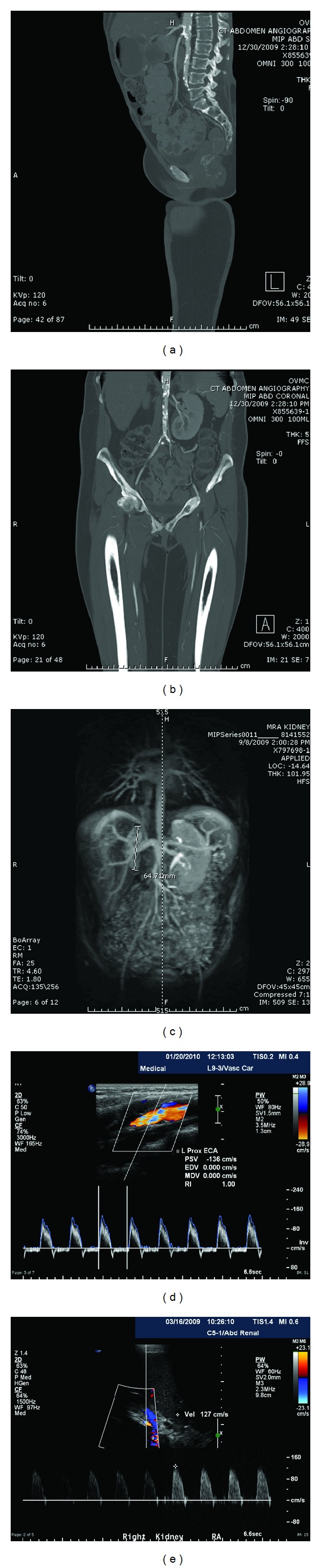
Images of patient J.S. and Takayasu's arteritis (a) Patient J.S. CT angiogram of abdomen and pelvis from 12/31/2009. Sagittal reconstruction showing severe calcifications along the abdominal aorta. (b) Patient J.S. CT angiogram of abdomen and pelvis from 12/31/2009. Coronal reconstructions showing severe abdominal aortic vessel calcification. (c) Patient J.S. MRA of abdomen and pelvis from 09/08/2009. 3D angiogram showing severe abdominal aortic calcifications along length of abdominal aorta, a small right kidney, moderate grade SMA stenosis, low signal in the right renal artery concerning for right renal artery stenosis or right renal artery occlusion, and narrowing of the bilateral iliac and femoral arteries (d) Patient J.S. Doppler flow of left external carotid artery from 01/20/2010. It shows a 50-69% stenosis, as well as notable intimal thickening all along the bilateral carotid arteries, including the left common carotid artery. (e) Renal ultrasound with Doppler from 03/16/2009. It shows an abnormally small right kidney measuring 6.1 cm, right renal artery with high resistance waveform and no diastolic flow suspicious for renal-artery occlusion versus stenosis.

The patient presented to nephrology clinic with no significant findings besides severe hypertension, for which she was managed on benazepril, isosorbide mononitrate, hydrochlorothiazide, and metoprolol. A renal ultrasound and duplex evaluation of the renal vessels at that time showed a normal-sized (11.4 cm) left kidney without sonographic evidence of renal artery stenosis. The left main renal artery was described as tortuous and diffusely narrow without focal stenosis. It also revealed an abnormally small (6.1 cm) right kidney with high resistance waveform with little to no diastolic flow (Figure 1).

The patient was lost to followup, but then presented herself six months later with hypertensive urgency. Laboratory workup revealed an elevated ESR of 23 mm/hr and a CRP of 9 (units). Laboratory studies including renal, hepatic, and hematological profiles were within normal limits. Secondary hypertension workup including ACTH, renin to aldosterone ratio, and catecholamines were negative. A renal MRA revealed asymmetric right kidney atrophy and delayed perfusion. The right renal artery was not well visualized, likely due to its markedly diminished caliber, confirming renal artery stenosis (Figure 1). Moderate grade SMA occlusion was also noted. The abdominal aorta's infrarenal segment, the bilateral common iliac arteries, the superior mesenteric artery, and proximal celiac axis were all noted to be stenotic (Figure 1). A second renal artery ultrasound with Doppler was ordered and this time showed right renal artery stenosis with abnormal wave form indicative of high resistance and no diastolic flow in the right renal artery (see Figure 1). 

The patient continued to deny any symptoms. On physical exam at that time, she was noted to have asymmetric brachial and radial pulses, a blood pressure difference greater than 10 mm Hg between arms, diminished dorsalis pedis pulses, and abdominal bruits. The patient was then started on oral prednisone which she did not tolerate and was switched to weekly methotrexate. 

Cardiac workup was negative, but a lower extremity arterial duplex ultrasound showed ankle brachial indices consistent with minimal to moderate ischemia and claudication on the right (ABI 0.63) with moderate to severe ischemia on the left (ABI 0.48). A CT angiogram of the abdomen and pelvis showed diffuse atherosclerotic changes involving the abdominal aorta confirming the MRA (Figure 1). Carotid ultrasound was sent to look for further manifestations of TA and found bilateral carotid stenosis with <50% stenosis in the right common carotid, and 50-69% stenosis on the left distal external carotid artery. Diffuse and bilateral carotid intimal thickening was noted all along both carotid arteries, including the left common carotid artery (Figure 1).

Given the extensive atherosclerotic lesions found on CT angiogram of the abdomen and pelvis, it was concluded that the patient may have some form of vasculitis and the patient's medical history was revisited. The patient remembered having had some type of lymphoma as a child, which was treated in an unknown manner. The patient said she had “a lot of X-rays,” but denied receiving radiation treatments. A diagnosis of TA was made after the aforementioned TA criteria and the patient's history were reviewed. The patient continues with oral weekly methotrexate and is being monitored closely by outpatient nephrology, cardiology, vascular surgery, interventional radiology, and rheumatology. For further details regarding patient J.S. and for a summary of her presentation see Table 1.

### 2.2. Case Number 2: Patient Y.P

Patient Y.P. is a 26-year-old female with no known past medical history who initially presented to a community health clinic for a routine physical. She had not seen a physician for many years and her only complaint was bilateral lower extremity cramping after 3 blocks of walking. She had unremarkable past medical, surgical, and social histories. She had no family history of hypertension and was not on any medications. 

**Figure 2:**
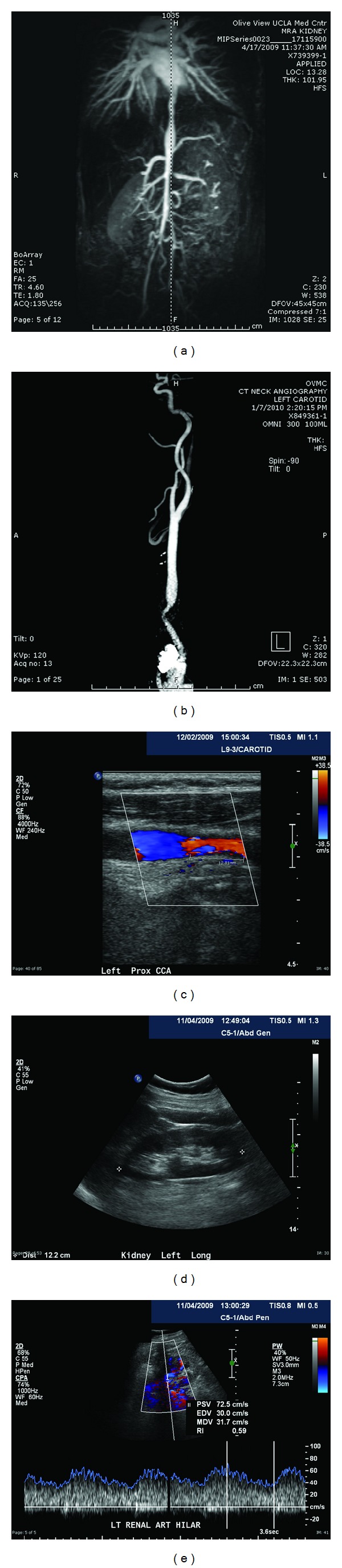
images of patient Y.P. and Takayasu's arteritis (a) Patient Y.P. MRA of abdomen and pelvis from 04/01/2009. 3D angiogram showing thoracic aortic stenosis at the level of the descending aorta and abdominal aortic stenosis. Stenosis of the left renal artery is also identified. (b) Patient Y.P. Carotid CT-Angiogram of the neck from 01/07/2010. 3D angiogram showing striking left common carotid artery stenosis. (c) Patient Y.P. Carotid Ultrasound with Doppler flow imaging from 12/02/2009. Figure shows a severe left common carotid artery stenosis of 70%. (d) Patient Y.P. renal ultrasound from 11/04/2009. Figure shows a normal sized left kidney measuring 12.2 cm. (e) Patient Y.P. renal ultrasound with Doppler flow imaging from 11/04/2009. Figure shows left renal artery stenosis by Doppler velocity measurement showing an abnormal resistive index of 0.59 and velocity of 182 cm/s fulfilling one of two criteria for left renal artery stenosis.

The patient's vitals were notable for elevated blood pressure, and her physical exam revealed a loud systolic murmur, audible even from the back. A routine PPD was placed and found to be positive so a chest X-ray was ordered. Her chest X-ray was clear of any infiltrates and she was diagnosed with latent tuberculosis and started on Isoniazid therapy. Over the next few weeks, the patient's blood pressure remained elevated despite multiple antihypertensive medication titrations. Her primary care provider from the community clinic ordered a MRA of the renal arteries to evaluate for secondary hypertension, which was abnormal for multiple sites of stenosis. She was then referred to Nephrology clinic at Olive View-UCLA. The MRA of the kidney showed a moderate grade stenosis of the left renal artery as well as segmental fusiform narrowing of the descending thoracic aorta, and infrarenal abdominal aorta, and narrowing of the right external iliac artery suspicious for high-grade stenosis. Segmental occlusion of the left common iliac artery, occlusion of the proximal superior mesenteric artery, and hypertrophied collaterals emanating from the inferior mesenteric and lumbar arteries were also noted (Figure 2). A renal ultrasound was ordered and showed elevated resistive indices and a high velocity (182 cm/s) in the left renal artery especially near the hilum. These findings helped confirm left renal artery stenosis (see Figure 2).

An MRA of the chest was ordered to evaluate the thoracic vessels. The thoracic MRA revealed focal stenosis and coarctation of the descending thoracic aorta with a 3 cm segment of narrowing in the proximal abdominal aorta. The MRA of the chest also showed decreased perfusion to the right upper lung suggestive of pulmonary artery stenosis; thus, an echocardiogram was ordered to evaluate for pulmonary hypertension, which revealed an increased PA pressure of 43 mmHg. The chest MRA also showed findings consistent with left carotid artery stenosis so a carotid ultrasound with Doppler was ordered and showed focal area of wall thickening and luminal narrowing with marked elevation of the velocity in the proximal level of the left mid common carotid artery (Figure 2). These findings were again seen on a neck CT angiogram that revealed an approximately 1.1 cm segment of severe narrowing of the left common carotid artery (Figure 2). In addition to the radiographic findings, her ESR and CRP were elevated at 21 and 13.4, respectively. All these findings taken together helped establish the diagnosis of TA.

Since her diagnosis, she has been followed by OVMC rheumatology, nephrology, and vascular surgery. She was started on prednisone by rheumatology with a normalization of the ESR and CRP to 13 and 6.6, respectively. She was continued on the Isoniazid therapy for the latent TB without any complications. The most challenging part of the management in this case has been the refractory hypertension requiring high doses of multiple antihypertensive agents. Her blood pressure remained elevated at routine clinic visits. For further details regarding patient Y.P. and for a summary of her presentation see Table 1.

### 2.3. Case Number 3: Patient M.R

 Patient M.R. is a 44-year-old female with type 2 diabetes, diagnosed with TA in 1990 at age 24. She presented with uncontrolled hypertension, and as a result of her TA, she had a nonfunctional right kidney secondary to right renal artery stenosis. She had no other pertinent medical, family, or social history, including no prior history of infection with *Mycobacterium Tuberculosis. *When she presented to the renal clinic, she was taking prednisone 10 mg once a day, atenolol 50 mg once a day and maxzide 37.5 mg/25 mg once a day for management. However, the patient remained routinely hypertensive on this regimen. Patient's physical exam was routinely negative for subclavian and carotid bruits. The patient had normal serum chemistry profiles, including electrolytes and creatinine. Her diabetes was diagnosed in 2005 after long-term steroid treatment for her TA. The patient's ESR remained high between 30 and 73 (mm/hr) during her time at OVMC. The patient's chest X-rays were immediately noted as abnormal with moderate to severe calcified atherosclerosis noted in the thoracic aorta. The patient's chest CT scans were ordered and in June of 2001, a CT angiogram of the chest revealed aortic atherosclerosis of the aortic arch, and a repeat CT scan in July 2002 also showed marked “chunk-like” aortic calcifications in the aortic arch. There was also a suggestion of right renal artery stenosis seen in the 2002 CT scan of the chest in the lower cuts of the scan. There was no evidence of subclavian artery stenosis in her imaging reports. A carotid duplex ultrasound was performed to rule out carotid stenosis in December 2002. The images showed minimal intimal thickening bilaterally, thickening of the right carotid system, and a severe stenosis of the left common carotid artery in the proximal, mid, and distal segment.

A captopril renogram was done in September 2002 suggesting right renal artery stenosis, decreased perfusion to the right kidney, and compensatory hypertrophy of the left kidney. In September 2003, an MRA of the abdomen and pelvis was done which confirmed high grade right renal artery stenosis versus occlusion and a small right kidney secondary to hypoperfusion. An enlarged left kidney was noted, likely due from compensatory hypertrophy. The superior mesenteric artery was noted as diminutive and the radiologists suggested a possible stenosis. The MRA also identified a diminutive proximal left superficial femoral artery. 

After the patient represented in 2003 with abdominal pain, an MRA of the abdomen was ordered and confirmed complete Superior Mesenteric Artery (SMA) stenosis. She had her treatment for a relapse of TA restarted with steroids, which had been tapered and stopped altogether in September 2003. Azathioprine was discontinued shortly thereafter in October 2003. For further details regarding patient M.R. and for a summary of her presentation see Table 1.

## 3. Discussion

### 3.1. Patient J.S. and Possible Exposure to XRT

In the first case there was initially some concern that the patient had concurrent radiation arteritis and TA. She denied history of previous radiation treatments, and the patient definitely met criteria for TA (see Table 1). The relationship between TA and radiation arteritis is not well described. However, there may be an increased risk of future radiation arteritis in a patient with preexisting collagen vascular disease. Lin et al. reported that a diagnosis of collagen vascular disease (rheumatoid arthritis, systemic lupus erythematosus, scleroderma, dermatomyositis, polymyositis, polymyalgia rheumatica, temporal arteritis, Wegener granulomatosis, ankylosing spondylitis, and mixed connective tissue disorders) may predispose to radiotherapy toxicity [12]. 

Reddy et al. described a patient with breast cancer treated with chemotherapy and radiotherapy who developed postirradiation morphea and subcutaneous polyarteritis nodosa [13]. Related phenomena following irradiation include postirradiation panniculitis and polyarteritis nodosa. Radiation may be responsible for inducing some of the pathogenic changes seen in autoimmune diseases. Concurrent TA and radiation arteritis is possible in this case though not explicitly proven. Autoimmune diseases can increase risk of radiation arteritis, but it is not known if the converse is true. It is important to note that the heavy calcifications are not usually observed in TA and could represent an atypical presentation of TA, radiation arteritis, or much less likely concurrent atherosclerotic vascular disease (ASVD) which would be atypical given the patient's age. 

### 3.2. Patient Y.P. and Linking TB with Autoimmune Diseases Like TA and UC

Case number two, patient Y.P., explored a more radiographically and clinically typical presentation of TA. The interesting link between TB and TA is evident in this case in that patient Y.P. was being treated for latent TB. It is interesting that TA has been linked serologically with an autoantibody that cross reacts 38 kDa protein from *Mycobacterium Tuberculosis *[5]. It is also notable that TA is more prevalent in TB endemic nations like India, Central America, South America, and Turkey. Genetics and specific HLA types may play a role in the association between TB and TA. This is also suggested by the link between UC and TA, as it is well known that UC may be related to a postinfectious reaction to an atypical pathogen in patients with specific HLA types. These disease associations are tantalizing clues that atypical infections, like tuberculosis, may be associated with an autoimmune sequellae such as TA and UC. This mechanism may be implicated in many other autoimmune diseases. The exact mechanisms are currently being studied in both UC patients and TA patients.

### 3.3. Patients M.R. and a Classical Example of TA

Patient M.R. presents a more typical example of TA. She had diffuse disease including bilateral carotid artery narrowing/stenosis and severe right renal artery stenosis. The patient also had severe atherosclerotic disease and demonstrated vascular calcifications. Atherosclerotic vascular disease of this magnitude is highly unlikely in this population of young females, and is almost certainly related to their underlying vasculitis, though it is not one of the published major or minor criteria of TA derived from Japanese populations. Her presentation with abdominal pain prompted increased clinical concern for mesenteric ischemia. The radiographic confirmation of SMA occlusion notes the importance of vigilance in this disease, as an active flare can occlude or result in aneurysmal dilatation of any medium or large artery in the body. The successful treatment of patient M.R. is actually atypical of relapsing TA. Usually half of TA patients have a benign course, and in those cases, TA presents as a monophasic illness in these patients that does not require immunosuppression. The other half, like M.R., require more intensive therapy with glucocorticoids and immunosuppressive agents like azathioprine [14]. The remission of patient M.R.'s symptoms for two years is fortuitous given her previous relapses.

### 3.4. Differences in Our Case Presentations and Classical TA in Asian Populations

The number of cases presented in this case report is small; only three patients of Hispanic descent with TA are described, but some major differences are noted. One difference already discussed is that TA is less common among the Hispanic population. Therefore, it is unusual to see three Hispanic patients with this disease (the only three Hispanic patients with TA in our clinic). Though there is some shared genetic ancestry between Asian and Hispanic populations as previously discussed in the introduction. 

The first major clinical difference noted is that none of our patients demonstrated subclavian stenoses, which are a major criterion and is a common feature in presentations of TA in traditional Asian populations. The second observation is that the link between TB and TA was established in population studies of TA patients in Central and South America, rather than among traditional populations with TA. This makes TB infection a risk factor for the development of TA that is different within the Hispanic population than among the Japanese population which has a much lower incidence of TB infection. The presence of concurrent vascular calcifications is an atypical radiographic feature of TA not typically described in Japanese populations. Also notable was the presence of medium to high grade SMA stenosis in two of our patients. While it is not unreasonable to expect stenosis of the branches of the descending aorta, this finding is not part of the published major or minor criteria of TA. Larger population studies could help identify other unique epidemiological, etiological, and pathophysiological manifestation in this population that may differ from the heavily studied Japanese population. Currently, the patterns of TA in Mexico shows that for the most part, TA case reports in that nation “follow disease manifestations in Asian nations” [7]. Further studies on other Central and South American immigrants in the United States may yield a similar conclusion or yield exciting new epidemiological patterns and pathophysiological connections between other diseases and TA.

## Figures and Tables

**Table 1:** Clinical summary of three Hispanic patients with Takayasu's arteritis.

Patient ID	Age/gender	Major criteria		Minor criteria									Other factors			
		RSS	LSS	ESR	HTN	CC tender	AR or AE	PA	L.CCA	BCT	Th.A	AA	TB	Rad	Calc.	UC
J.S.	37 F	(+bruits)	(+bruits)	**23**	+	−	trace AR	−	+	−	−	+	−	? +	+	−
Y.P.	27 F	−	−	**21**	+	−	−	−	+	−	+	+	+	−	−	−
M.R.	44 F	−	−	**50–74**	+	−	−	−	+	−	+	+	−	−	+	−

(Age: note age is age at time of diagnosis of Takayasu's arteritis) Major Criteria: LSS: left subclavian artery stenosis, RSS: right subclavian artery stenosis minor criteria: AA/AE: abdominal aortic lesion/aortic ectasia, AR: aortic regurgitation, BCT: brachiocehpalic trunk arterial lesion, CC tender: common carotid tenderness, ESR: erythrocyte sedimenation rate, HTN: hypertension, L.CCA: left common carotid artery lesion, PA: pulmonary artery lesion, Th.A: thoracic aortic lesion other factors: Calc: calcifications, Rad: radiation therapy exposure, TB: tuberculosis, UC: ulcerative colitis, ?: questionable, + present, − absent.
